# Enhancing phagocytic capacity in layer chickens: a one health approach to sustainable production through improved disease resistance and reduced antibiotic dependence

**DOI:** 10.3389/fvets.2026.1802884

**Published:** 2026-05-14

**Authors:** Shunqi Liu, Jingchao Cao, Shuqi Wang, Shiyu Qi, Yue Zhao, Shoulong Deng, Zhichao Sun, Wendi Zhou, Kun Yu, Yitong Yuan, Zhengxing Lian

**Affiliations:** 1 Beijing Key Laboratory for Animal Genetic Improvement, National Engineering Laboratory for Animal Breeding, Key Laboratory of Animal Genetics and Breeding of the Ministry of Agriculture, College of Animal Science and Technology, China Agricultural University, Beijing, China; 2 National Animal Husbandry Services, Beijing, China; 3 College of Animal Husbandry and Veterinary Medicine, Jinzhou Medical University, Jinzhou, Liaoning, China; 4 Shandong Aijia Poultry and Livestock Co., Ltd., Liaocheng, Shandong, China; 5 Department of Laboratory Animal Center, Shanxi Medical University, Taiyuan, Shanxi, China

**Keywords:** antibiotic-free rearing, disease resistance, layer, monocyte/macrophage, phagocytosis product

## Abstract

**Background:**

Reducing antibiotic dependence in poultry production is critical to mitigating antimicrobial resistance (AMR). Selective breeding for stronger innate immunity may provide a sustainable alternative under a One Health framework. This study investigates whether enhancing monocyte/macrophage phagocytic capacity in layer chickens could improve disease resistance without compromising production performance under antibiotic-free conditions.

**Methods:**

Phagocytic capacity was assessed using multiple assays. Phagocytosis product (PP) was measured using the MTT-HCT-8 method, pHrodo-labeled *E. coli*, and FITC-labeled *S. pullorum*, whereas the phagocytic index (PI) was determined using *O. aries* erythrocytes. Chickens were divergently selected into high (HPPG) and low (LPPG) phagocytosis product groups based on monocyte/macrophage phagocytic capacity at 16 weeks of age (wk). Production traits, immune-related gene expression, oxidative stress-related markers, and pathogen-clearance ability were compared between groups using Student's *t*-test.

**Results:**

PP measured by the MTT-HCT-8 method was significantly correlated with PP or PI using the other methods (*P* < 0.05, *R*^2^ > 0.70). HPPG hens showed significantly higher laying rates at 40 and 60 weeks and greater hatch weight than LPPG hens (*P* < 0.05), whereas fertility, hatchability, or semen quality did not differ between groups. HPPG birds exhibited higher interferon-gamma receptor (IFNGR) and Toll-like receptor 2 (TLR2) expression and, after *S. pullorum* challenge, showed reduced tumor necrosis factor α (TNF-α) and increased microtubule-associated protein 1 light chain 3 alpha (LC3A) expression (*P* < 0.05), indicating enhanced pathogen clearance and autophagy without exacerbating oxidative stress or inflammation.

**Conclusion:**

Selecting for proper high phagocytic capacity (with a preliminary estimated PP range of 1.58–2.74) improves resistance in layer chickens reared antibiotic-free without reducing productivity and exacerbating oxidative stress or inflammation. These findings support phagocytosis-based phenotyping as a potential balanced-breeding strategy consistent with One Health goals for sustainable poultry production.

## 1 Introduction

For the demand of safer, more humanitarian, eco-friendly, and commercial products (1–3), the layer industry is facing the challenges of improving egg quality, keeping hens' reproduction, discovering indigenous chickens' novel commercial relative traits, and heightening layers' immune ability and stress resistance, especially under an antibiotic-free environment (4–7). These challenges align with the “One Health” initiative: a holistic framework that recognizes the inextricable linkages between human, animal, and environmental health (8). Thus, the central challenge for sustainable, antibiotic-reduced production is to identify and leverage immune phenotypes that confer robust protection while being genetically compatible with optimal production performance. Consequently, a One Health-aligned strategy necessitates a “balanced breeding” approach that concurrently improves disease resistance and maintains productivity (9–12).

Early breeding programs for disease resistance in chickens mainly targeted specific diseases, such as Marek's disease (13), and extensively investigated the role of the major histocompatibility complex (14, 15). A hypothesis regarding the conflict between and within productive and disease-resistant traits in livestock breeding is widely supported (16–20). Still, with the evolution of technology and the expansion of our cognition, immunity has been proven not to be significantly correlated with production on phenotypes (21) and to advance alongside the production benefits (22).

Monocytes/macrophages play important roles in innate immunity (23–26). Some previous researches aim at chickens' monocytes/macrophages' phagocytic capacity and demonstrate that the ability can be measured by phagocytosis product (PP) and phagocytosis index (PI) (27–30) and the high capacity selection benefits flocks' disease resistance-related traits, such as survival rate, IgG titer and the amount of CD4+ T cells in serum and IgY titer in egg white (27–29), but the productive traits, such as laying rate and body weight, perform conflict between the two groups of different chicken flocks (27–29). In addition, the quantitative relationships among commonly used phagocytosis indicators, including PP, PI, and phagocytic percentage, remain unclear, which limits the broader use of monocyte/macrophage phagocytic capacity as a breeding phenotype (31, 32).

During immune activation, monocytes and macrophages generate oxidative reactions as part of host defense (33, 34). This process is physiologically necessary for pathogen killing, but an excessive oxidative response can damage cells and tissues, disrupt immune homeostasis, and ultimately impair animal health and productive performance (35–37), such as fertility (34). Importantly, NF-κB-related signaling links these processes: it not only drives immune and pro-inflammatory responses, but also participates in oxidative stress-related regulation (37–39). Thus, although selection for high phagocytic capacity may enhance the clearance of pathogens and other exogenous stimuli, it is still unclear whether such selection also amplifies pro-inflammatory or oxidative responses to a degree that could produce side effects. This question is especially relevant for the One-health breeding strategy that aims to improve disease resistance while maintaining production in antibiotic-free environments.

This study evaluated whether breeding for higher monocyte/macrophage phagocytic capacity in Nongda No. 3 dwarf chickens could enhance disease resistance without inducing excessive inflammatory or oxidative responses. Using the oxidative stress-related and MyD88-NFκB-related inflammatory markers for basal cellular status and pathogen-challenged immune responses, comparisons were conducted between chickens of HPPG and LPPG. Also, by integrating production trait assessments with multiple phagocytosis assay methodologies, the proper phagocytic capacity range for breeding would be preliminarily defined. The goal of this research aims to provide a practical breeding parameter that aligns with One Health objectives by promoting disease resistance and reducing reliance on antibiotics in poultry production.

## 2 Materials and methods

### 2.1 Chickens, growth conditions, trait collection and breeding strategy

Closed-bred Nongda No. 3 dwarf chickens were divergently selected from previously published research (27), and the generation comes to G2, with a total of 1,029 individuals. All animal experiments were approved by the Animal Care and Use Committee of China Agricultural University and conducted in compliance with the National Research Council's Guide for the Care and Use of Laboratory Animals (Permit Number: AW60203202-1-2). Chickens grow up in the conditions of Chinese northern rural barn with three-tier cage systems, natural lighting, and antibiotic-free, drug-free management. Vaccination was administered according to the standard schedule. The chickens were raised under identical environmental conditions, with feed and water provided *ad libitum*. One bird per cage after 16 weeks of age (wk) and each bird was accurately identified by its cage number. Roosters were randomly sold from 90 days of age onward by farm supervisors, and breeding roosters were sold at 45 wk; breeding hens were sold at 73 wk.

**Figure 1:**
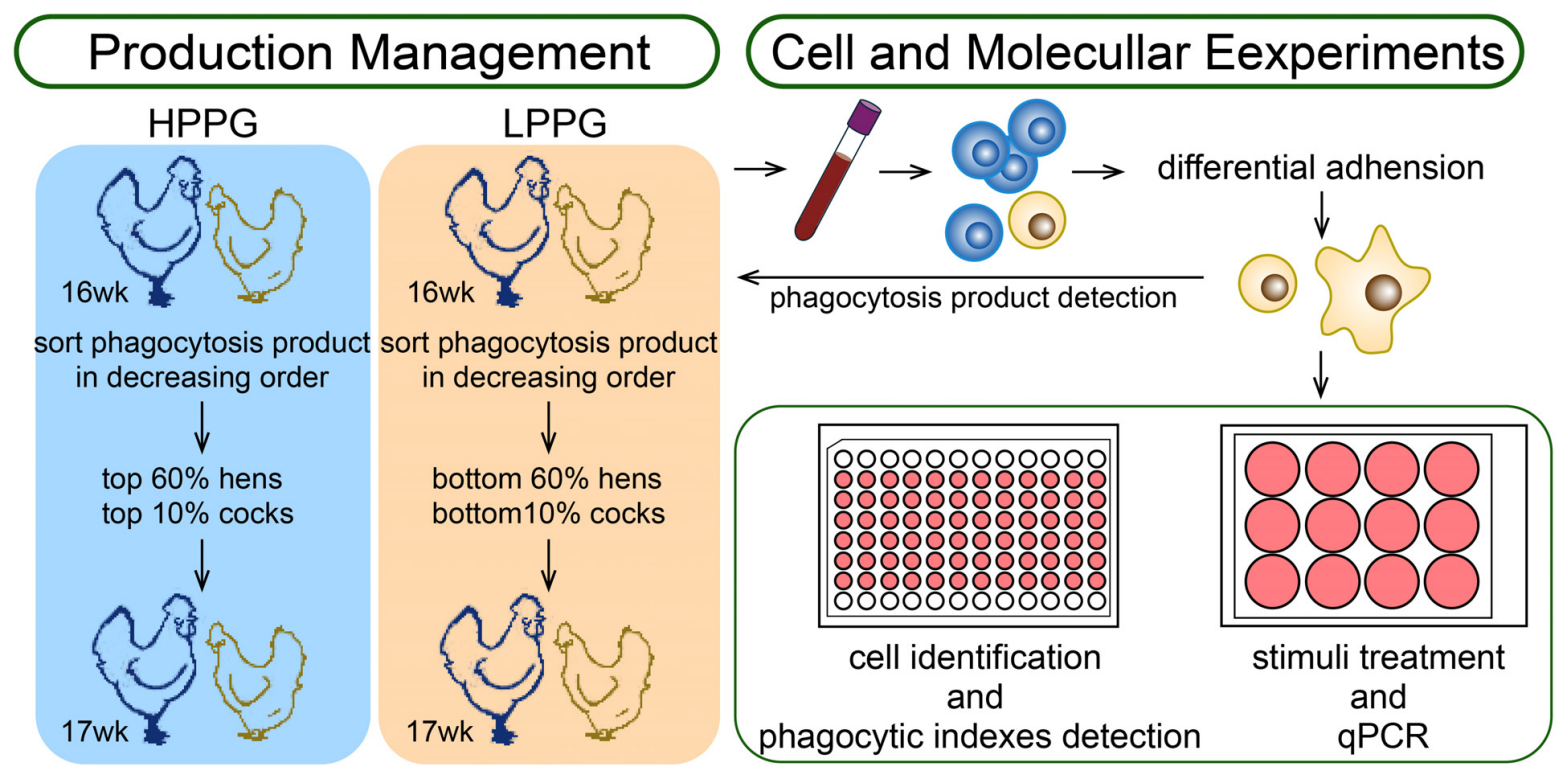
The illustration of two workflows: production management **(left)** and molecular and cellular experiments **(right)**. Divergently selected flocks by PP of monocytes/macrophages. Monocytes were separated by differential centrifugation after peripheral blood collection from wings' veins and the differential adhesion method, partly differentiated into macrophages after culturing, and finally, for PP assay, cell identification, phagocytic-related treatment, and qPCR. The blue cells stand for lymphocytes, and the brown cells represent monocytes and macrophages.

The breeding strategy and farm management scheme were based on PP measured by the MTT-HCT-8 method. This assay, including preparation of the MTT-HCT-8 suspension and its final cell density (2 × 10^8^/ml), was previously described by Yuan et al. (2018) (27). Divergent selection was performed at 17 wk according to the PP measured at 16 wk. Chickens were assigned to the high phagocytosis product group (HPPG) and the low phagocytosis product group (LPPG), specifically on the basis of PP determined by the MTT-HCT-8 assay. Within the previously established HPPG and LPPG populations (27), chickens were culled based on thresholds determined by a selection pressure of 60% for hens and 10% for roosters. Specifically, within each group, birds were ranked in descending order based on PP measured at 16 wk. In the HPPG, the top 60% of hens and the top 10% of roosters were retained for breeding. Conversely, in the LPPG, the bottom 60% of hens and the bottom 10% of roosters were retained for breeding. In total, PP was measured in 158 roosters and 374 hens. The breeding strategy is summarized in Figure 1.

Roosters began ejaculation training through dorsal massage at 30 wk, with semen collection performed for three consecutive days, after which the roosters were rested for 4 days. Semen quality has been assessed during 34–36 wk by ejaculation rate, semen volume, sperm vitality, sperm motility, and sperm morphological abnormality. Semen collection was performed on eight HPPG and seven LPPG roosters. Each bird underwent 15 scheduled collection attempts, and samples from the same rooster were treated as technical replicates. After that, the ejaculation training was conducted as before, and artificial insemination was conducted at 38 wk when hens were fertilized for consecutive 3 days and eggs were collected for the following 4 days. Fertility rate and hatchability were calculated at 40 and 42 wk. Hatch weight was collected. Laying performance collection was scheduled on 22–24, 60 and 72 wk according to the grandparent stocks' comparison in Yuan's research (27). The mortality was recorded throughout the whole production stage of each flock.

### 2.2 Cells separation and culture conditions

Chickens' peripheral blood monocytes were obtained from the wings' veins using the Chicken Peripheral Blood Mononuclear Cell Isolation Solution Kit (LDS1088C, TBD, Tianjin, China), according to the manufacturer's instructions. Cells were cultured at 37 °C, 5% CO_2_, in RPMI 1640 medium (11875119, gibco, MA, USA) containing 10% FBS (10099, gibco, MA, USA) for 1 h to separate monocytes (adherence on the bottom) and lymphocytes (easy to refloat in the medium). In subsequent experiments, the mononuclear cell isolation and culture methods followed the protocol described above. After separation from lymphocytes, monocytes/macrophages were cultured for 59 h, with a change of medium at 48 h, till the confluence around 75%, for the following treatments. When further experimental procedures involve lymphocytes, fresh lymphocytes must be isolated again following the aforementioned separation protocol for subsequent processing. Unless otherwise specified, the same mononuclear-cell isolation and culture procedures were used throughout this study.

### 2.3 Monocytes/macrophages identification—gimsa and KUL01 staining

Monocytes/macrophages were stained by the Rapid Giemsa staining kit (E607314, Sangon Biotech, Shanghai, China) according to its manufacturer's protocol. For immunostaining, chicken's monocytes and macrophages marker KUL01 (40) was used for staining as follows. After 60 h of culture in 96-well plates, cells were fixed with Immunol Staining Fix Solution (P0098–100 ml, Beyotime, Shanghai, China) for 20 min at room temperature. Cells were then washed with D-PBS (D1040, Solarbio, Beijing, China) and Immunol Staining Wash Buffer (P0106, Beyotime, Shanghai, China) for 5 min, fixed with Immunol Staining Fix Solution for 40 min, and blocked with Immunol Staining Blocking Buffer (P0102, Beyotime, Shanghai, China) for 40 min at room temperature. Then, samples were washed three times by D-PBS and incubated with a 100 μL volume of a 1:400 dilution of KUL01 primary antibody (sc-52603, SANTA CRUZ BIOTECHNOLOGY, CA, USA) for 6 h at 4 °C. After washing, cells were incubated with a 100 μL of 1:500 dilution of secondary antibody (ZB-2306, ZSGB-BIO, Beijing, China) for 2 h at room temperature, washed three times by D-PBS, and examined under a confocal fluorescence microscope.

### 2.4 Phagocytosis product assay

Mononuclear cells were separated from 0.5 ml wings' veins peripheral blood and cultured in 96-well plates (3599, Corning, OH, USA), with six parallel wells per individual. Half of the wells were assigned to the Phagocytosis Group and half to the Control Group (Supplementary Figure S1). The medium was replaced after 1 h of culture (41). At this stage, monocytes and early macrophage-like derivatives remained adherent, whereas lymphocytes were largely non-adherent, enabling physical separation of the monocyte/macrophage population. Thereafter, the medium was changed every 30 h. At 61 h, the phagocytosis group received 20 μL of MTT-HCT-8 suspension for 10 h, whereas the control group received 20 μL MTT (M8180-1 g, Solarbio, Beijing, China; dissolved in D-PBS to 5 mg/L) for 4 h. Media were removed as completely as possible, the Phagocytosis Group wells were gently washed twice with D-PBS and cells marked Formazan were dissolved in 150 μL DMSO (D4540, sigma, MI, USA), with shacking for 10 min. Absorbance was measured at 570 nm and 630 nm in a microplate reader (Synergy HT, BioTek, VT, USA). The absorbance of the Phagocytosis Group reflects the uptake of MTT-HCT-8, and that of the Control Group stands for the living monocytes/macrophages count. All steps after addition of MTT-HCT-8 were performed in the dark. PP was calculated using the formula below:


$$\begin{array}{l} Phagocytosis\;product=\;engulfed\;indicators\;÷\;living\\ \;cells=\;Phagocytosis\;group\;OD\;÷\;control\;group\;OD\\ \;=\;Phagocytosis\;group\;\left(OD_{570\;nm}\;-\;OD_{630\;nm}\right)÷\;control\;group\;\left(OD_{570\;nm}\;-\;OD_{630\;nm}\right) \end{array}$$

Unless otherwise specified, the “phagocytosis product” or “PP” stands for monocytes/macrophages challenged with MTT-HCT-8. Phagocytosis product was also detected by treated with pHrodo-labeled *E. coli* (pHrodo-*E. coli*, P35361, Invitrogen, CA, USA, diluted into 100 μg/ml) for 40 min and FITC-labeled *S. pullorum* (FITC-*S. pullorum*, the suspension preparation was supported in Supplementary materials, bacteria density of the mother liquor was modulated to 1 × 10^4^ cfu/μL with D-PBS) for 1 h at a multiplicity of infection (MOI) of 20:1. The optimal stimulation times for these fluorophore-conjugated bacterial indicators are shown in Supplementary Figure S2, and the optimal stimulation time for *O. aries* erythrocytes is shown in Supplementary Figure S3. When fluorescence-labeled inactivated bacteria were used to determine PP, one unit consisting of three pixels in ImageJ was counted as one indicator.

### 2.5 Phagocytic indexes assay

Monocytes/macrophages were incubated with *O. aries*' erythrocytes prepared as described in Li et, al.'s research (2018) (42), cell density of the mother liquor was modulated to 1 × 10^7^/ml with D-PBS) for 1 h, then washed three times with D-PBS, fixed by Immunol Staining Fix Solution, imaged under a microscope, and quantified to detect PI and phagocytic percentage. Erythrocytes were added to monocytes/macrophages at a 20:1 ratio to assess phagocytic capacity. The PI and phagocytic percentage were calculated by the formulas shown below:


$$\begin{array}{l} Phagocytosis\;Percentage\;=\;the\;count\;of\;phagocytic\;cells\;\\ \;÷\;total\;cells\\ \;Phagocytic\;Index\;=\;engulfed\;indicators\;÷\;phagocytic\;cells \end{array}$$

### 2.6 *S. pullorum* clearance assay

*S. pullorum* [cvcc533, China Center for the Preservation and Management of Veterinary Microorganisms, the same bacterial strain as the previous research, which Li et al., 2008 (29) and Ma et al., 2010 (28)] was cultured in 5 ml LB medium for 24 h, then seeded on solid LB medium for culturing 6 h for bacteria activity checking. Well-active *S. pullorum* was counted by bacterial colonies seeded on solid LB medium after gradient dilution, and the bacterial solution was diluent into 1 × 10^6^ cfu/μL with D-PBS.

Mononuclear cells were seeded in 12-well cell culture plates (3,336, Coring, OH, USA) at a density of 2.5 × 10^6^ cells/1,000 ml/well. After 60 h of culturing, monocytes/macrophages were challenged with *S. pullorum* at an MOI of 40:1 for 60 min. After infection, cells were washed twice with D-PBS containing 2% penicillin-streptomycin (15070063, gibco, MA, USA), each washing lasting 30 s, and lysed with 0.25% Triton-100 (X100-100 ml, Sigma-Aldrich, MI, USA). The lysate was subsequently washed twice with D-PBS. The precipitated bacteria were resuspended in a liquid LB medium and seeded to a solid LB medium for culturing for 18 h, and bacterial colonies were counted for differential analysis to determine the differences in the ability to eliminate *S. pullorum* between HPPG and LPPG chickens under the antibiotic-free rearing.

### 2.7 Cells harvest, RNA extraction, cDNA synthesis and qRT-PCR

Acquired 5 ml peripheral blood, gained mononuclear cells, seeded them in 12-well plates, and harvested monocytes/macrophages and lymphocytes after corresponding treatments. Total RNA was extracted by the EASYspin RNA micro Kit (RN56, Aidlab, Beijing, China) following the manufacturer's instructions. First-strand cDNA was synthesized by the PrimeScript RT reagent Kit (RR047A, Takara, Tokyo, Japan), and qPCR was performed with Taq ProUniversal SYBR qPCR Master Mix (Q712, Vazyme, Jiangsu, China). Glycer-aldehyde-3-phosphate dehydrogenase (GAPDH) was used as the internal control. This study chose hens in well-health, and laying rate greater than 75% as the samples for this experiment. All primers used for qPCR analyses are listed in Supplementary Table S1.

### 2.8 Statistical analysis

All data were presented as mean ± standard error. The relative gene expression levels were calculated using the 2^−ΔΔCt^ method. The comparisons on PP, productive traits, and *S. pullorum* clearance between HPPG and LPPG were analyzed by *t*-test on R (Lucent Technologies, version 4.4.2). The correlation and linear regression analysis between different phagocytic capacity methods were performed on R. Significance levels were established as follows: *P* < 0.05 (significant, marked^*^), *P* < 0.01 (highly significant, marked^**^), and *P* < 0.001 (extremely significant, marked^***^). Non-significant results (*P* ≥ 0.05) were presented with their exact *P*-values to allow for effect size interpretation. Data visualization was performed using GraphPad Prism (GraphPad Software, version 9).

Before the *t*-test, the Shapiro–Wilk test was employed to verify whether the data within each group followed a normal distribution. ① If the data were normally distributed, a homogeneity test for variance would be performed. In the case of homoscedasticity, the homoscedasticity assumption *t*-test would be applied; else, a heteroscedasticity assumption *t*-test would be conducted. ② If either group exhibited a deviation from normality, data of both the HPPG and LPPG would undergo a log (x + 1) transformation, after which the normality of the within-group data distribution would be reassessed. ③ If the transformed data for both groups conformed to a normal distribution, the variance homogeneity test would be applied again. Once the transformed data of the two groups revealed homoscedasticity, a homoscedasticity assumption *t*-test would be engaged; else, the heteroscedasticity assumption *t*-test would be used. ④ If the transformed data of either group did not follow a normal distribution, the non-parametric test–the Wilcoxon rank-sum test, would be conducted on the raw data. The flowchart for statistical analysis procedure is illustrated in Supplementary Figure S4. All plots were generated from raw data.

## 3 Results

### 3.1 Phagocytosis assays are highly correlated and can be standardized

**Figure 2:**
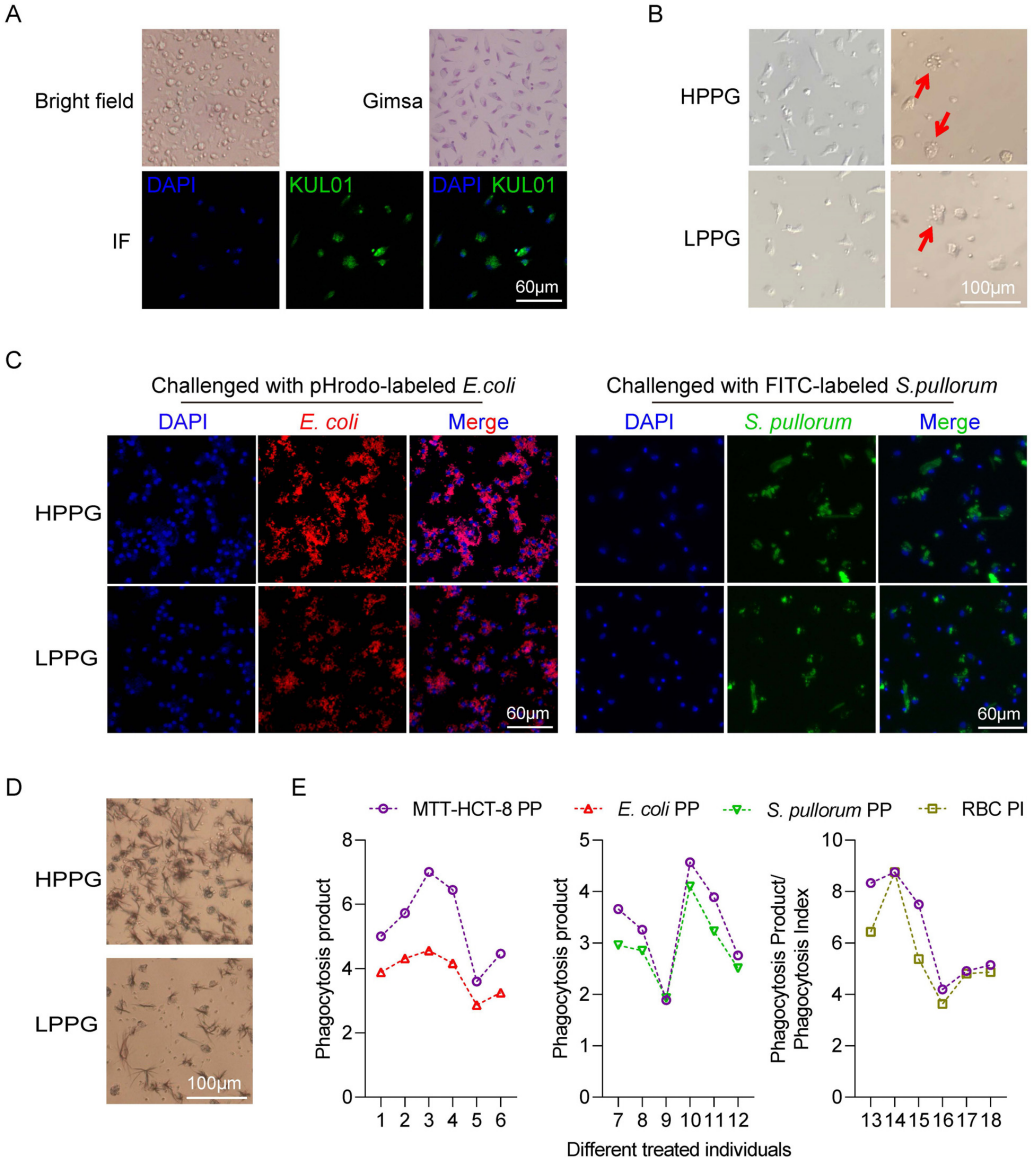
Phagocytic capacity detected by MTT-HCT-8 method positively correlated to that detected by other methods. **(A)** Identification of monocytes/macrophages by Gimsa stain and KUL01 immunofluorescence. **(B)** The monocytes/macrophages before *O. aries*' erythrocytes treated **(left)** and the uptake of *O. aries*' erythrocytes by monocytes/macrophages **(right)**. Red arrows demarcate the engulfment of erythrocytes by monocytes/macrophagocytes. **(C)** The uptake of pHrodo-*E. coli* **(left)** and FITC-*S. pullorum* **(right)** by monocytes/macrophages. **(D)** The uptake of MTT-HCT-8 by monocytes/macrophages. **(E)** The regression of phagocytosis product of monocytes/macrophages detected by MTT-HCT-8 to that detected by fluorescence-stained dead *E. coli* **(left)** and *S. pullorum* **(middle)**, and to phagocytosis index detected b*y O. aries*' erythrocytes **(right)**.

The identification of isolated cells as monocytes/macrophages was confirmed through Giemsa staining and KUL01 immunostaining in Figure 2A. The uptakes of different phagocytosis indicators, such as *O. aries*' erythrocytes, pHrodo-*E. coli*, FITC-*S. pullorum* and MTT-HCT-8, were exhibited in Figures 2B–D. The PP detected by the MTT-HCT-8 method was significantly regressive to that detected by pHrodo-*E. coli* and FITC-*S. pullorum* and PI were detected by *O. aries*' erythrocytes (Figure 2E), and regression formulas were displayed in Table 1. This consistency across different indicators suggests that the MTT-HCT-8-based PP assay reliably reflects the general phagocytic function of monocytes/macrophages.

**Table 1:** The linear regression significance between the MTT-HCT-8 assay and other phagocytosis assay methods.

Methods of phagocytic capacity detection	PP detected by MTT-HCT-8 (Sample size: six biological replicates within three technical replicates in each biological replicate)		
	***P***	**Adjusted *R^2^***	**The liner regression formula**
PI detected by *O. aries*' erythrocytes	0.022	0.71	*y* = 0.74x + 0.44
PP detected by pHrodo-*E. coli*	0.004	0.87	*y* = 0.49x + 1.21
PP detected by FITC-*S. pullorum*	0.003	0.89	*y* = 0.80x + 0.49

According to previous studies (27–29), the linear regression analysis revealed that 0.90–2.74 might be a proper range that can benefit both production and disease resistance traits in hens, and for roosters, the proper range might be 0.61–4.40.

### 3.2 Selection based on phagocytosis product does not compromise production traits

**Figure 3:**
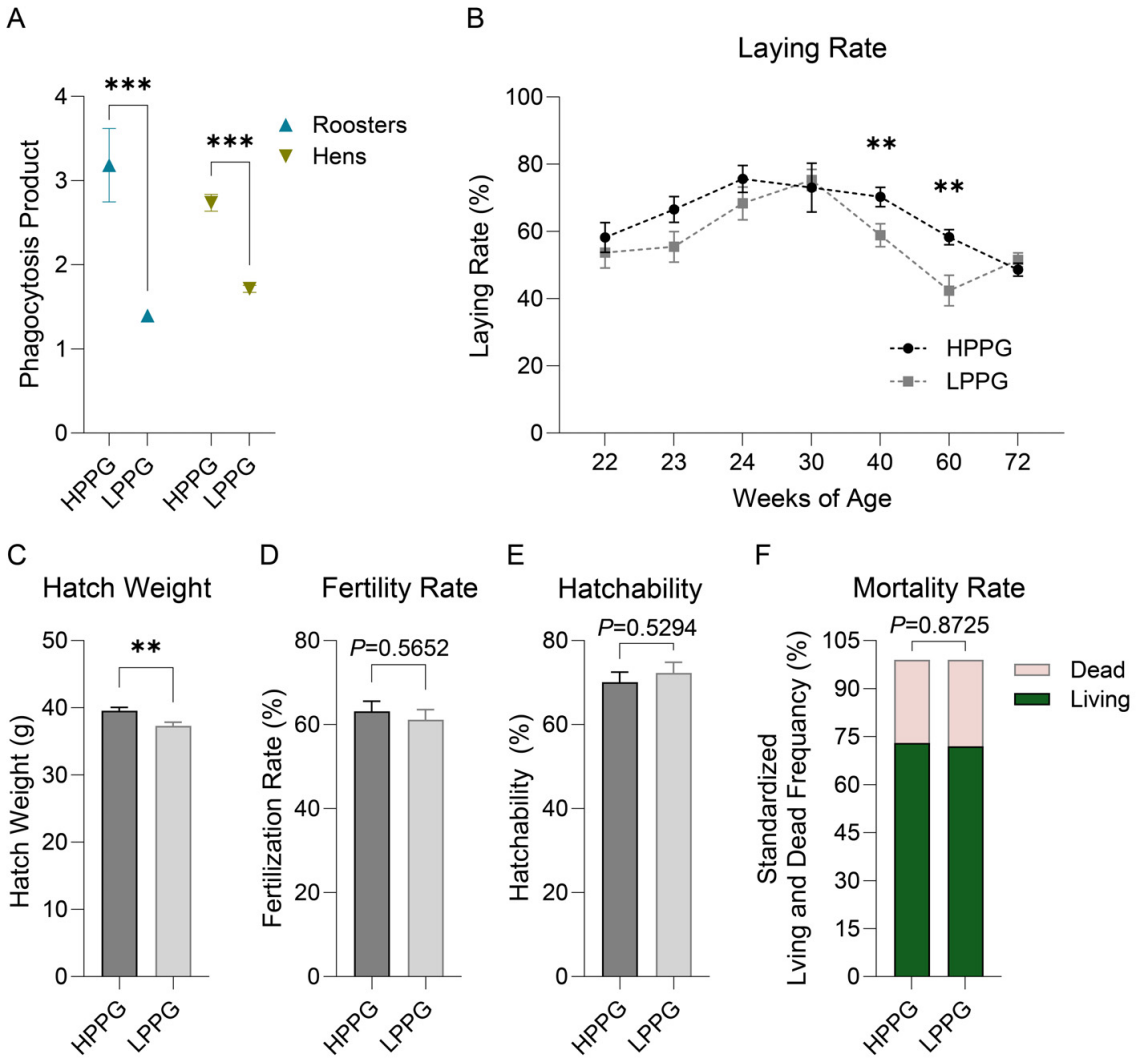
Comparison of commercial-related traits between HPPG and LPPG flocks. **(A)** The PP between HPPG and LPPG with divergent selection. **(B)** Laying rate of HPPG and LPPG. **(C)** Hatching weight of HPPG and LPPG. **(D)** Fertility rate of HPPG and LPPG. **(E)** Offspring hatching rate HPPG and LPPG. **(F)** 16–72 wk mortality rate of HPPG and LPPG. ** indicates *P* < 0.01, *** indicates *P* < 0.001.

After selection, the LPPG comprised seven roosters and 92 hens, and the PP average of roosters was 1.39, and that of hens was 1.71; the HPPG comprised seven roosters and 78 hens, and the PP average of roosters was 3.18, and that of hens was 2.74 (Figure 3A). The difference between the PP of HPPG and LPPG was significant.

The laying rates of HPPG at 40 wk and 60 wk were 70.25 ± 24.25 % and 58.28 ± 18.13 %, respectively, and were significantly higher than those of LPPG at the same weeks of age, which were 58.85 ± 28.05 % and 42.40 ± 25.39%, respectively (Figure 3B). The hatch weight of HPPG was 39.97 ± 0.48 g, significantly higher than that of LPPG, which was 37.30 ± 0.55 g (Figure 3C). While the fertility rate (Figure 3D), hatching rate of in-group offspring (Figure 3E) and mortality rate of 16–72 wk (Figure 3F) had no differences between the two groups.

No significant between-group differences were observed in semen quality in roosters (Table 2).

**Table 2:** The semen quality of the two groups' roosters.

Semen quality parameters	HPPG	LPPG	*P*-value
Ejaculation rate (%)	53.85 ± 0.14	35.71 ± 13.29	0.36
Semen volume (μL)	270.00 ± 53.85	364.28 ± 112.71	0.52
Sperm vitality rate (%)	90.04 ± 0.22	89.75 ± 0.36	0.49
Sperm motility rate (%)	85.00 ± 0.05	79.83 ± 0.04	0.46
Sperm morphological abnormality (%)	2.75 ± 0.73	3.93 ± 0.76	0.28

### 3.3 Chickens of HPPG exhibit enhanced immune readiness without oxidative stress

The flocks divergently selected by PP revealed some differences in production-related traits, like previous research (27–29). However, it remained unclear whether the cellular response behavior of pro-oxidant and pro-inflammatory functions of monocytes/macrophages and lymphocytes was altered, thereby affecting systemic immunity, growth, and production. In this section, key immune response-related receptors and pathways, pro-inflammatory cytokines, and pro-oxidant factors in both monocytes/macrophages cultured for 61 h and freshly isolated monocytes and lymphocytes were examined by qPCR, to reveal the impact of divergent PP selection on the pro-oxidant and pro-inflammatory capacities of these cells.

**Figure 4:**
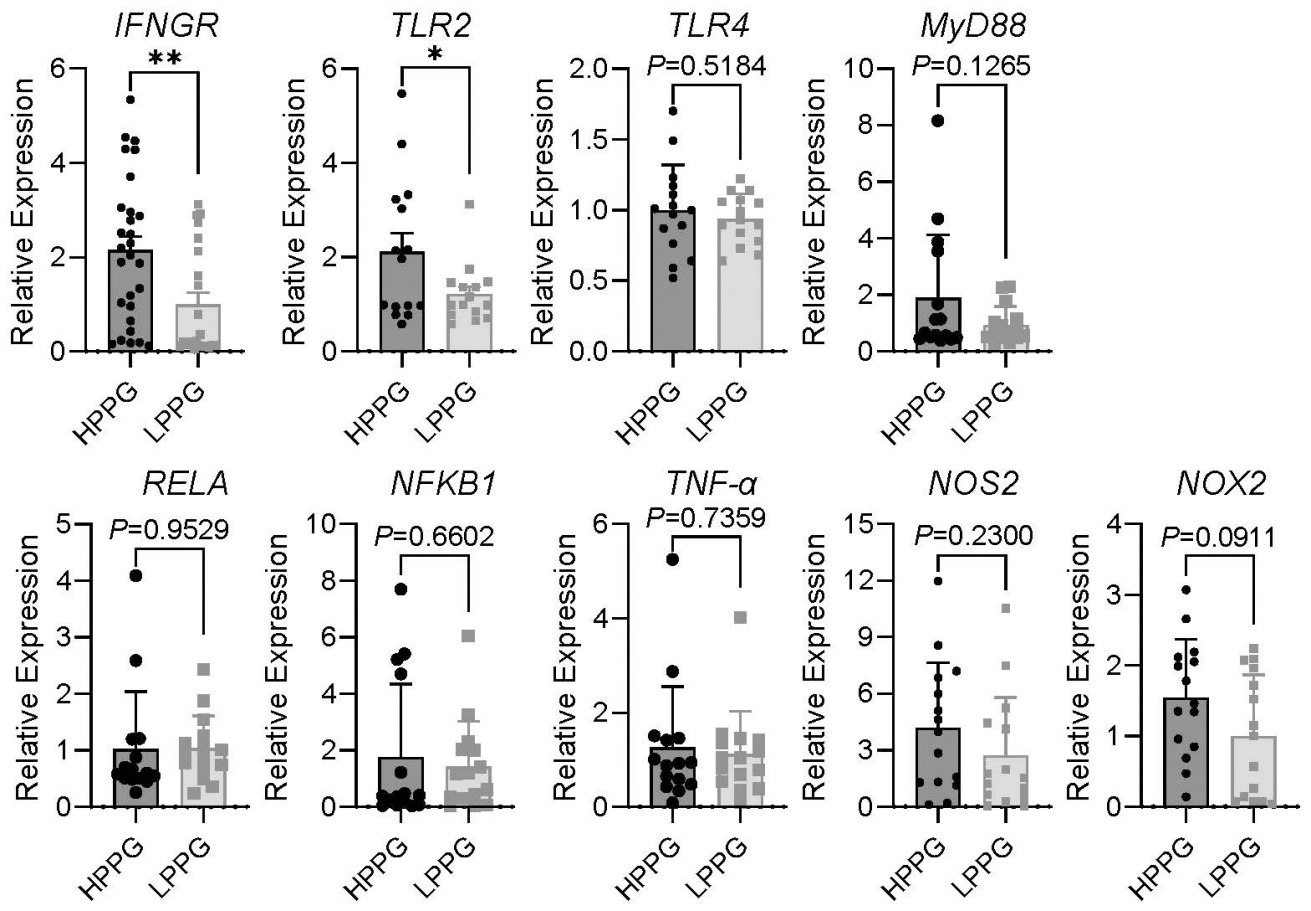
qPCR assay of inflammation and oxidation-related genes in monocytes/macrophages, with 61-h culturing. Comparison results of relative expression of IFNGR, TLR2, TLR4, MyD88, RELA, NFKB1, TNF-α, NOS2, and NOX2 between HPPG and LPPG are displayed. * indicates *P* < 0.05, ** indicates *P* < 0.01.

**Figure 5:**
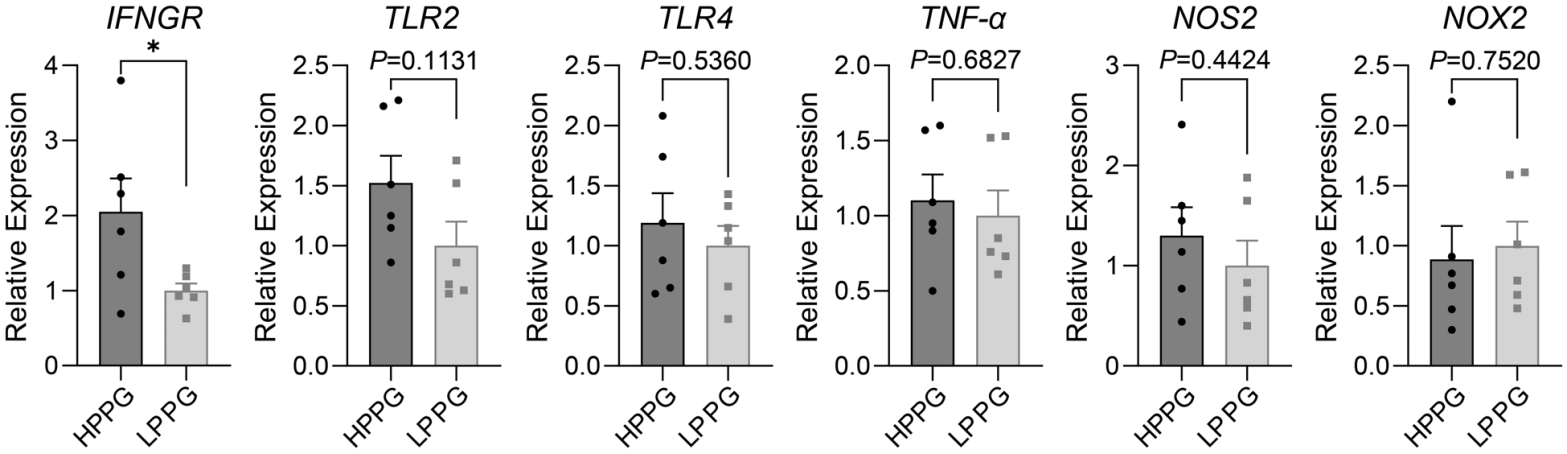
qPCR assay of inflammation and oxidation-related genes in monocytes/macrophages, with 1-h culturing. Comparison results of relative expression of IFNGR, TLR2, TLR4, TNF-α, NOS2, NOX2 between HPPG and LPPG are displayed. * indicates *P* < 0.05.

Chickens of HPPG showed enhanced immune readiness without elevated oxidative or inflammatory tone. The monocytes/macrophages of HPPG exhibited higher expression of IFN-γ receptor (IFNGR) and Toll-like receptor 2 (TLR2) after being cultured for 61 h than those of LPPG, and no differences in the MyD88-NF-κB (RELA and NFKB1)-TNF-α (TNFA) inflammation signaling pathway, nor the pro-oxidants (NOS2 and NOX2; Figure 4). Consistent with this, in monocytes/macrophages that were immediately subjected to RNA extraction and reverse transcription after lymphocytes removal by 1-h culturing of mononuclear cells, the expression of IFNG of HPPG was higher than that of LPPG by qPCR (Figure 5).

**Figure 6:**
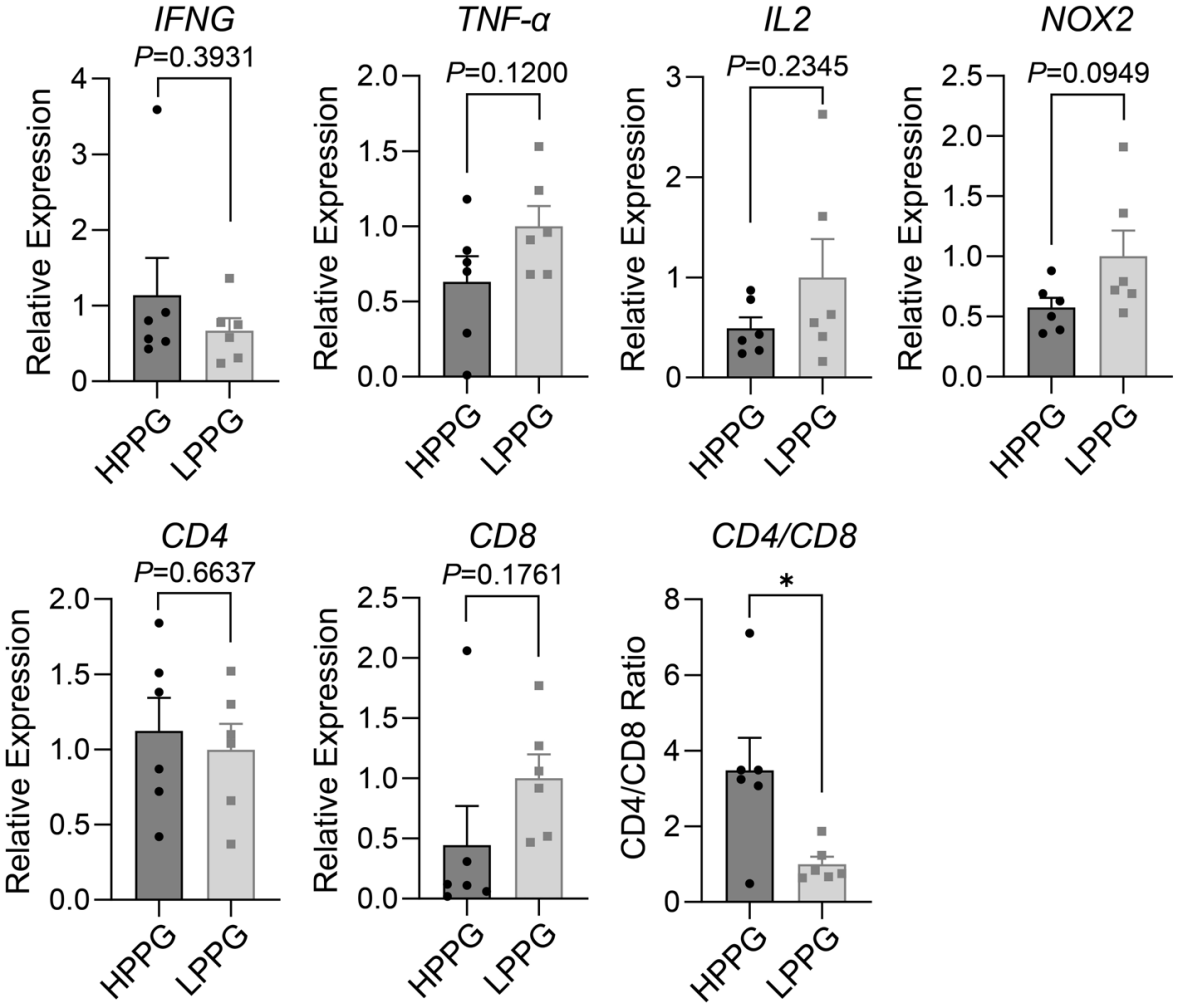
qPCR assay of inflammation and oxidation-related genes in lymphocytes, with 1-h culturing. Comparison results of relative expression levels of IFNG, TNF-α, IL2, NOX2, CD4, CD8, and CD4/CD8 ratio between HPPG and LPPG are displayed. * indicates *P* < 0.05.

In lymphocytes cultured for 1 h, the inflammation and oxidation-related genes had no significant differences between HPPG and LPPG, but the ratio of CD4 to CD8 in HPPG was higher than that in LPPG (Figure 6). HPPG chickens exhibited a primed but balanced immune state.

### 3.4 Macrophages of HPPG chickens show stronger *S. pullorum* clearance with modulated inflammation

**Figure 7:**
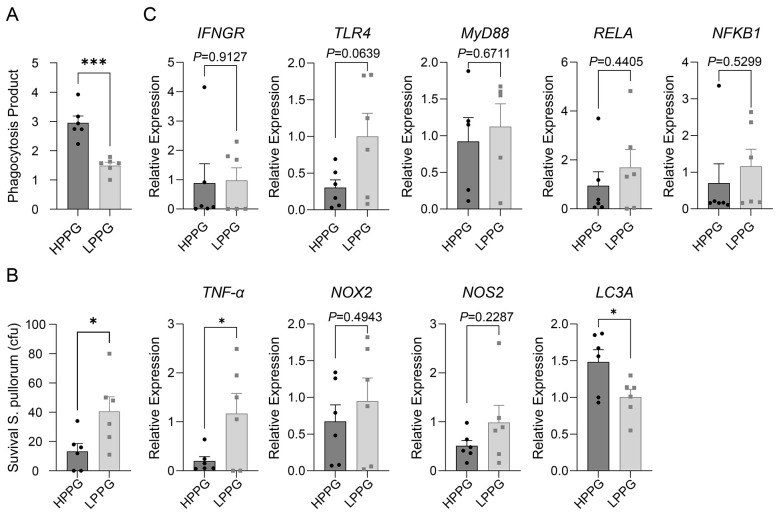
The expression of inflammation and oxidation-related genes after monocytes/macrophages challenged with *S. pullorum* for 1 h. **(A)** The PP detected by FITC-*S. pullorum*. **(B**) The clearance capacity of HPPG and LPPG monocytes/macrophages. **(C)** The qPCR assay of *S. pullorum*-related inflammatory and oxidation mediators. * indicates *P* < 0.05, *** indicates *P* < 0.001.

After culturing for 61 h and challenged by *S. pullorum*, the PP was detected by FITC-*S. pullorum* of HPPG was significantly higher than that of LPPG (Figure 7A). The HPPG exhibited a higher *Salmonella* clearance efficiency than the LPPG (Figure 7B). The expression of TNFA in LPPG was higher than that of HPPG, while the expression of LC3A in HPPG was higher than that of LPPG (Figure 7C). Upon bacterial challenge, they showed enhanced clearance and a tendency toward autophagy over excessive inflammation.

These findings supported the feasibility of using phagocytic phenotype as a selection criterion for breeding chickens with improved disease resistance in antibiotic-free systems.

## 4 Discussion

Previous research demonstrates that the phagocytosis product can stand for phagocytic ability (27–29). Horseradish peroxidase-labeled *E. coli* (29) and MTT-HCT-8 (27) are reported to be used for phagocytosis product assay, and heterologous erythrocytes are reported to be used for phagocytosis index assay (28, 30). This study reveals that the PP detected by the MTT-HCT-8 method is significantly regressive to PP detected by pHrodo-*E. coli* and FITC-*S. pullorum* and PI detected by *O. aries*' erythrocytes. That means the phagocytic capacity assay in this study, based on the MTT-HCT-8 method, can mathematically correspond to other parameters detected by other methods in previous studies. The reason why MTT-HCT-8 method was adopted in this study is that this method can perform high-throughput phenotyping of phagocytic capacity by phagocytosis product, and MTT-HCT-8, which can be well stored at 4 °C (27), is a kind of stable, cheap, and easy-to-prepare indicator.

Using regression-based conversion together with previously reported data (28, 29), we estimated that a PP interval of 0.90–2.74 at 16 wk may be advantageous for hens in terms of both production and disease resistance. In the present study, PP in HPPG hens ranged from 1.58 to 5.31, with the interval from 1.58 to 2.74 accounting for 50% of the HPPG population. This group showed better laying performance at 40 and 60 wk and stronger resistance to *S. pullorum*, consistent with previous reports (28, 30). For roosters, the HPPG PP interval was 2.78–5.32, whereas the previously suggested suitable range was 0.61–4.40 (27–29), covering 86% of the roosters in this study.

No significant differences appeared between roosters of HPPG and LPPG on reproductive traits (Figures 3C, D and Table 2). Notably, the laying rate and semen volume displayed high standard errors (Figure 3C and Table 2), which differed from the observation in well-established commercial poultry productions (43, 44). Based on the research design and production management, first, we propose several possible reasons for the large standard error in laying rate. ① The G2 flock was derived from the lineage in the research of Yuan et al. (27, 45), where the standard error of laying rate was high. Thus, the performance of the G2 flock might be inherited from the G0 and G1 populations. ② After PP measurement, the number of hens in each group in the study was only one quarter of that in the G0 study (27). The reduced sample size likely increased sampling error around the group mean, resulting in a larger standard error and lower statistical precision. This is an important statistical explanation for the large variation in laying rate. ③ From G0 to G2, divergent selection only depended on PP, for principle of single-variable control, to evaluate the effect of PP variation on production-related traits. However, laying rate is not a qualitative trait but a complex quantitative trait. Its heritability varies across laying stages or ages, without a consistent increasing or decreasing trend. Therefore, in the absence of continuous selection pressure specifically targeting laying rate, the trait is unlikely to remain phenotypically stable across generations (46). A similar pattern was observed in semen volume, which also revealed a relatively large standard error. ① Semen volume already displayed a high standard error in the progenitor population (27). ② By the markedly reduced sample size in this study, where the number of roosters per group after PP measurement was only one quarter of that in the G0 study (27). ③ However, unlike laying rate, the G2 breeding roosters showed numerically better mean values for semen volume, sperm vitality rate, and sperm morphological abnormality than the G0 breeding roosters, although these differences were not statistically significant. Because semen collection and artificial insemination procedures are similar across the poultry industry, this situation suggests that the genetic parameters of semen quality traits would be relatively comparable under different management conditions. Previous studies have reported that the heritability of semen volume in chickens was approximately 0.28, indicating moderate heritability, whereas sperm vitality rate and sperm morphological abnormality have heritability estimates of approximately 0.85 and 0.60, respectively, indicating relatively high heritability (47). That is, in the absence of selection pressure, sperm vitality rate and sperm morphological abnormality may decline less across generations than semen volume. Nevertheless, in the discussion of semen volume standard error in this study, the most important point is sample size. Although technical replicates were relatively abundant, the number of biological replicates was limited from the perspective of statistical analysis of production traits.

So, in future studies evaluating the feasibility of phagocytic index as a breeding indicator, it will be necessary to increase the sample size of roosters and to perform a more systematic assessment of semen quality-related traits; or taking the advantage of the extremely high proportion of hens rearing in the egg industry, the next exploration phase, divergently select hens based on PP, while comprehensively documenting their laying performance to establish a more precise and commercially or clinically relevant reference range for the PP distribution in laying hens at 16 wk.

By 1-h culture, the mononuclear cells from blood can be separated into monocytes/macrophages (attached on the bottom) and lymphocytes (in the medium) (48, 49), and the continuous culturing helps monocytes differentiate into macrophages (50). Peripheral blood-derived monocytes are categorizable into macrophage-differentiable and non-macrophage-differentiable subsets (51). Besides, the research of Li et al. proves that monocytes/macrophages, which is identified with CD14^+^ and CD11b^+^, can be gained by culturing for 50 h (42). Therefore, this study proposes that 61-h-cultured monocyte/macrophages consist of: ① monocytes incapable of differentiating into macrophages, ② monocytes capable of differentiating into macrophages, and ③ macrophages (MΦ) (52). Conducting phagocytic capacity assays at this time point provides a comprehensive assessment of innate immunity cells, which also have the pathogen clearance capacity, when exposed to exogenous stimuli.

The qPCR results of 61-h cultured monocytes/macrophages demonstrate that HPPG display higher IFNGR and TLR2 expression than LPPG (Figure 4). IFNGR is reported to be highly expressed in the pituitaries of hens with 500 ml interferon-γ (IFNG, 100 U/ml) treated for 24 h, as is the expression of follicle-stimulating hormone (FSH) (45). Also, IFNGR, the receptor of IFNG, is an important receptor for macrophages polarizing in adaptive immune response (53–55). The previous studies on phagocytic capacity in chickens show that flocks with strong phagocytosis capacity have higher CD4^+^/CD8^+^ T cell ratio, higher IgG titer after vaccine and higher IgY concentration in egg yolk and egg white (27–29). Given the synergistic relationship between IFNGR, IFNG and FSH, as well as the association between high phagocytic selection and adaptive immunity observed in previous studies, this study prioritized IFNG as the primary indicator and performed qPCR analysis on the HPPG and LPPG groups. Due to the result of IFNG differently expressed between 22 hens of HPPG and 19 hens of LPPG, which are in well-health with a laying rate exceeding 75% at 50 wk, we limited the sample size to 15 per group to ensure that subsequent multiple target genes can be tested in a balanced and random design. TLR2, a general bacterial recognition receptor in avian species, can mediate a response to microbial stimuli (56), and its high expression in HPPG supports that the results of the PP assay reflect the broad-spectrum, non-antigen-specific phagocytic capacity of monocytes/macrophages.

The qPCR results of 1-h cultured monocytes/macrophages and T cells stand for the effect on the expression of target genes or physiological and biochemical processes by PP assay (Figures 5, **6**). The expression of IFNGR in monocytes/macrophages and the CD4/CD8 ratio in T cells are higher in HPPG, in accordance with previous research (29, 45). besides, the qPCR results with no significant differences between HPPG and LPPG, demonstrate that the PP selection doesn't influence oxidation and inflammation. Thus, PP selection had no significant impact on fundamental cellular metabolic processes.

Miyomo et al. also analyzed the genetic and phenotypic correlations between feed efficiency, immunity, and production traits in indigenous Kenyan chickens and found that immune traits, such as IgM and IgG titers (the former as an indicator reflecting innate immune and the latter reflecting adapted immune), had significant negative genetic correlations with egg production traits, including egg weight and number, but no significant phenotypic correlations between immune traits and production traits (21). These results demonstrated that combining proper management, rather than negatively affecting production traits, focusing on immune-related traits may benefit flocks' survival rate and strengthen disease resistance (21, 28, 29). This point can also be reflected in this study. Nevertheless, that HPPG monocytes/macrophages express a higher level of IFNG and HPPG T cells have a higher CD4^+^/CD8^+^ ratio reveals the high PP selection within the experimental range of this research, neither breaks redox homeostasis nor elevates pro-inflammatory cytokines, yet improves the sensitivity of the adapted immune response.

After being challenged with *S. pullorum* for 1 h, TNFA was expressed higher in monocytes/macrophages of LPPG, but the PP and the cleansing ability and LC3 expression of HPPG were higher and stronger in those of HPPG (Figure 7). That also reflects that the PP selection influences innate and adaptive immune response sensitivity and during the early phase of immune response (28, 29), when the gene expression of oxidative stress (NOX2 and NOS2) showed no significant differences (Figure 7), the monocytes/macrophages terminate inflammatory responses more rapidly and proceed to the autophagy stage, thereby mitigating inflammation-induced damage to cells or tissues (57, 58).

HPPG selection confers dual benefits: at the production level, it heightens flock hatch weight and laying rates; at the individual level, it mitigates inflammatory storms in monocytes/macrophages triggered by pathogens and oxidants while facilitating the transition to adaptive immune responses. However, the positive correlation between phagocytic capacity detected by different methods suggests the existence of unexplored, non-specific regulatory mechanisms governing phagocytic behavior. The more accurate range of PP for production and the mechanisms behind the PP regulation need to be further explored.

Our findings demonstrate that selective breeding for enhanced phagocytic capacity offers a viable pathway to reduce antibiotic dependence in layer chickens. The identified PP range supports both improved production traits and strengthened innate immunity, without inducing chronic inflammation or oxidative stress. This approach aligns with One Health principles by promoting animal health through natural resistance mechanisms, thereby reducing the need for antimicrobials and mitigating AMR risks. Future studies should explore the molecular regulators of phagocytosis and validate this strategy in diverse genetic lines and production systems. Implementing phagocytosis-based selection in breeding programs could advance sustainable poultry production while contributing to global efforts against AMR.

## 5 Conclusion

**Figure 8:**
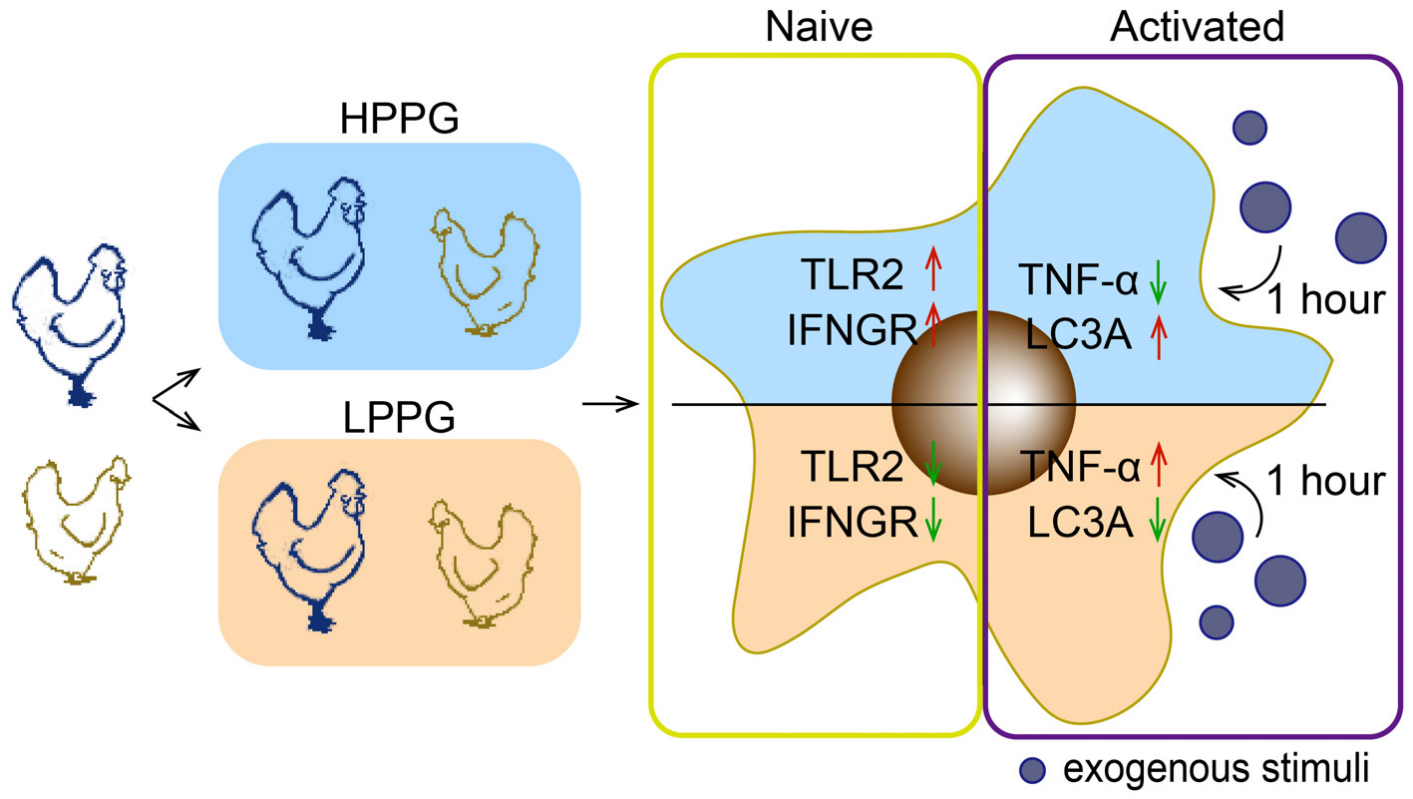
Graphical representation of transcriptional alterations in monocytes/macrophages of divergently PP-selected layer flocks. In an antibiotic-free rearing environment. HPPG selection helps monocytes/macrophages respond rapidly from inflammation to autophagy and reduce *TNFA* expression when facing pathogens and oxidants. The yellow and purple frames respectively denote the unstimulated and stimulated states, showcasing differentially expressed gene markers of immunity, oxidative stress, and inflammation in monocytes/macrophages between HPPG and LPPG.

In summary, the MTT-HCT-8 method for PP assay can indicate phagocytic capacity. Chicken flocks with proper high phagocytosis products can benefit production and disease resistance in an antibiotic-free rearing environment. While a preliminary suitable PP range of 1.58–2.74 was identified, more robust and validated ranges warrant additional research. At the immune level, compared to LPPG, HPPG selection helps monocytes/macrophages respond rapidly from inflammation to autophagy and reduce TNFA expression when facing pathogens without exacerbating oxidative stress (Figure 8).

## Data Availability

The raw data supporting the conclusions of this article will be made available by the authors, without undue reservation.
