# Supplementary material for: Enhancing phagocytic capacity in layer chickens: a one health approach to sustainable production through improved disease resistance and reduced antibiotic dependence
Source: Front Vet Sci. 2026 May 14;13:1802884. doi: 10.3389/fvets.2026.1802884 (PMC13215808; doi:10.3389/fvets.2026.1802884)
Supplement: Supplementary file 1 [file Table_1.DOCX]

Supplementary Material

# Supplementary Data

Supplementary Material should be uploaded separately on submission. Please include any supplementary data, figures and/or tables.

Supplementary material is not typeset so please ensure that all information is clearly presented, the appropriate caption is included in the file and not in the manuscript, and that the style conforms to the rest of the article.

## FITC-labeled *S. pullorum* Preparation

*S. pullorum* (China Center for the Preservation and Management of Veterinary Microorganisms, cvcc533, the same bacterial strain as the previous research, which Li et al., 2008, and Ma et al., 2010) within 0.5 ml Bacterial Cryopreservation Solution (D0391, Beyotime, Shanghai, China) was thawed and cultured in 5 ml LB medium for 24 h. The bacteria were then seeded on the solid LB medium and incubated for 6 h. A single bacterial colony was selected, and the process was repeated to ensure a population of actively growing bacteria with the same genetic background. Then, one bacterial colony was cultured with FITC-D-Lys (CS3304, G-CLONE, Beijing, China) for 24 h, following the manufacturer’s dyeing instructions. The bacteria were harvested in D-PBS after being washed. A 10 μL aliquot of the well-mixed suspension was stained with DAPI and diluted to 1 mL after washing twice. A 1 μL dilution sample was analysed to calculate FITC dyeing efficiency under the confocal microscope. 100 mL well-mixed fluorescence-marked bacterial suspension is used to be diluted into 1/1000, 1/5000, and 1/10000 densities and seeded on solid LB medium, respectively, for 6h to calculate the density and modulate the concentration of FITC-labeled *S. pullorum* to 1000 cfu/μL with D-PBS.

# Supplementary Figures and Tables

## Supplementary Figures

Figure S1 Layout of peripheral blood monocytes/macrophages’ phagocytosis product testing.

the Phagocytosis Group

the Control Group

Each column stands for one sample, the up three lines are challenged with indicators, and the down three lines are treated with MTT to reflect cell quantity. Every well contains 100 μL mononuclear cells-1640 suspension and needs to exchange medium on culturing first 1h and then per 24 h.


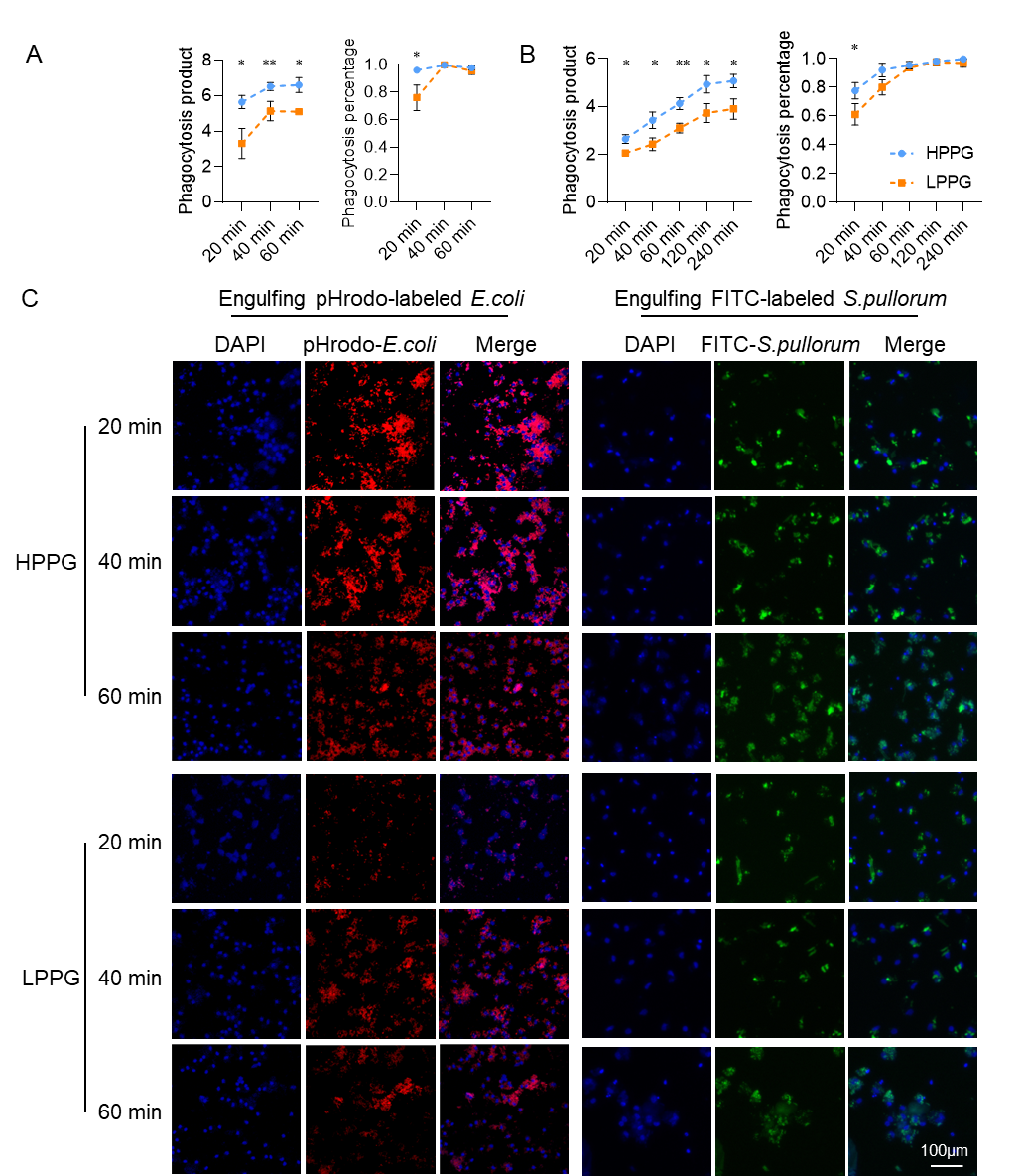


**Figure S2** Pre-experiment of monocytes/macrophages challenged with fluorescence-labeled dead bacteria. (A) The phagocytosis product and phagocytosis percentage of *E. coli* and (B) *S. pullorum*. (C) Monocytes/macrophages are challenged with pHrodo-*E. coli* and FITC-*S. pullorum* for different times —20 min, 40 min, and 60 min. Views were captured by fluorescence microscope.


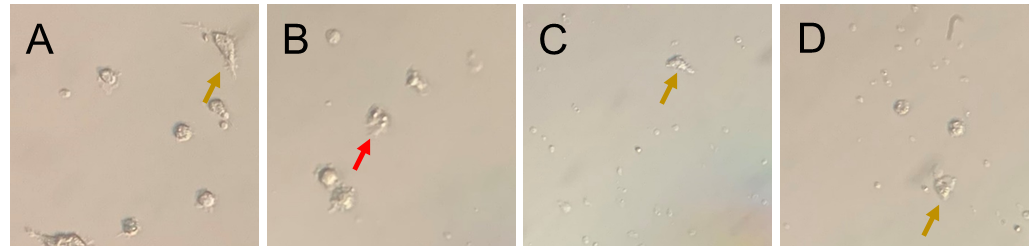


**Figure S3** Pre-experiment of monocytes/macrophages challenged with *O. aries*’ erythrocytes. (A) and (B) Monocytes/macrophages are challenged with *O. aries*’ erythrocytes for 1 hours. (C) and (D) Monocytes/macrophages are challenged with *O. aries*’ erythrocytes for 2 hours. Golden arrows refer to the unclear engulfing situation, and the red arrow refers to the clear engulfing situation.


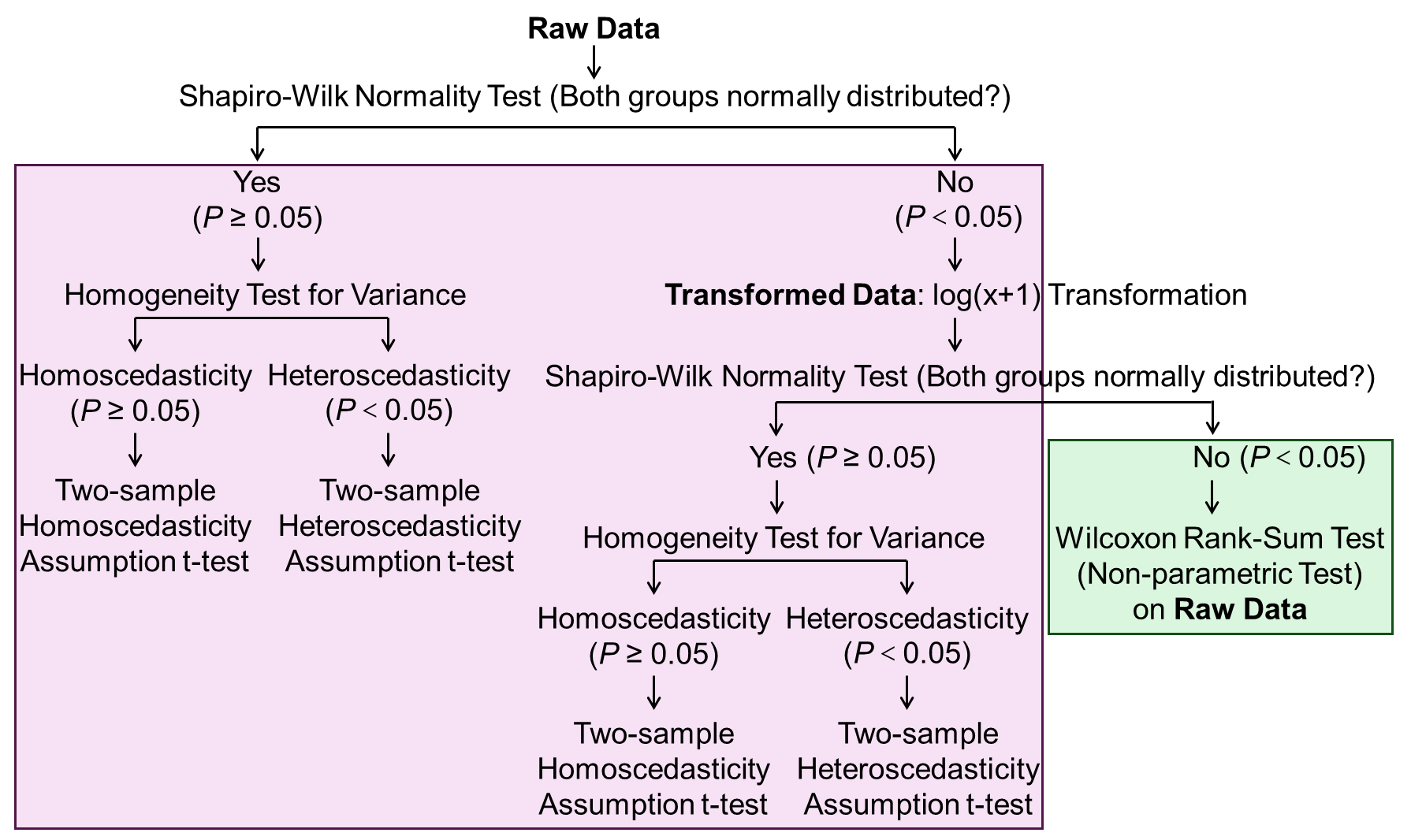


**Figure S4** Flowchart for Statistical Analysis Procedure of Raw Data. The purple box marks the workflow of a parametric test, and the green box marks the workflow of a non-parametric test.

## Supplementary Tables

**Table S1** Primers for qPCR (degenerate, if have variants)

| Gene | Primers (5’ →3’ ) | |
| --- | --- | --- |
| GAPDH | forward | TCGGAGTCAACGGATTTGGC |
|  | reverse | CCGTTCTCAGCCTTGACAGT |
| IFNGR | forward | GAACAACCATCAGCGAGGCG |
|  | reverse | CCAGCACCTCTAACTGAGGC |
| TLR2 | forward | AGCACTGTGAAGCACATTAAAGC |
|  | reverse | CAAGAGGTGGAAGGCTTGTG |
| TLR4 | forward | TGACCTACCCATCGGACACT |
|  | reverse | CTCAGGGCATCAAGGTCTCC |
| MyD88 | forward | AGAAAGAAGGTGTCGGAGGATG |
|  | reverse | GAATCAGCCGCTTGAGACGA |
| RELA | forward | CCCACCATCCGCGTAAACC |
|  | reverse | GGGGTTGTTATTGGTGCGGA |
| NFKB1 | forward | AAGGCAGATATACCAGGAGGAC |
|  | reverse | TTTGTGATTGTAAAGTTCACCTGTC |
| TNFA (TNF-α) | forward | GGGGTTGTTATTGGTGCGGA |
|  | reverse | GCCACTAGGAGCAGACATGA |
| NOS2 | forward | CCACTCATTCTCCAAGCAAAC |
|  | reverse | AACAGCAGGCAGAGCATACC |
| NOX2 | forward | GGATGAAACTCAGGCCACTC |
|  | reverse | GTGTACTCCTCGTGGGTCAG |
| IFNG | forward | GGCCAAGCTCCCGATGAA |
|  | reverse | AGTTGAGCACAGGAGGTCAT |
| IL2 | forward | GCAGTGTTACCTGGGAGAAGT |
|  | reverse | CGGTGTGATTTAGACCCGTAAGAC |
| CD4 | forward | CCCACTCACAACCCCATCTC |
|  | reverse | AGCGTTCCTCCTCAAAACCTAA |
| CD8 | forward | GCCTGGGGCTCTGCTGT |
|  | reverse | ATCCAGGAGACACCATTGTCG |
| LC3A | forward | TCCAAACAAAATCCCGGTCATCA |
|  | reverse | GTCTCCTGGGAAGCGTAGAC |
